# Intergenerational knowledge management in a cutting-edge Israeli industry: Visions and challenges

**DOI:** 10.1371/journal.pone.0269945

**Published:** 2022-07-08

**Authors:** Sigal Kordova, Orly Or, Arriel Benis

**Affiliations:** 1 Department of Industrial Engineering and Management, Faculty of Engineering, Ariel University, Ariel, Israel; 2 Faculty of Industrial Engineering and Technology Management, HIT-Holon Institute of Technology, Holon, Israel; Sri Eshwar College of Engineering, INDIA

## Abstract

Knowledge management is a multifaceted, complex, end-to-end organizational process dealing with collecting and using data, information, and knowledge generated by a group of individuals. The current study examines the changes required in companies’ quality systems to enhance intergenerational learning and knowledge retention. Our primary objective was to understand the factors that influence the development of an organizational culture encouraging innovation, knowledge sharing, organizational learning, openness, and providing opportunities to create up-to-date knowledge. We collected the viewpoints and needs of industry professionals by using interviews and a survey. Then, we analyzed the factors that influence knowledge management quality and transfer between workforce generations. The professionals’ primary goal is to introduce, integrate, and improve knowledge in their organization. Their second goal is to facilitate knowledge sharing and transfer between workforce generations. Improving transgenerational knowledge sharing and reducing the loss of knowledge are challenges for all industries. A cutting-edge industry such as the defense field deals with sensitive data, and knowledge management is a strategic need in a competitive context. Quality management standards propose guidelines for developing and enhancing the overall knowledge-related processes. However, implementing them requires a shift in the corporate culture team. Organizational knowledge resilience must be developed by involving the workforce in implementing knowledge management systems.

## Introduction

### Background

Organizations are in constant motion in a dynamic, global, and competitive environment. They need to reinvent themselves continuously to remain relevant and successful in their field. Knowledge is a critical resource for maintaining a sustainable competitive advantage [1, 2]. Like other resources, knowledge must be managed appropriately in the immediate and long terms. Knowledge management is generally a structured, consistent, methodical process designed to create and disseminate an organization’s information and innovative ideas [3, 4]. It enables the development of an organizational culture that encourages innovation and cultivates an environment that welcomes knowledge sharing, organizational learning, and openness while providing opportunities for producing new knowledge [5, 6]. These knowledge management products improve work processes and business reactivity to change, reduce organizational costs, and enhance learning from experience [7, 8].

For an organization that wants to be a learning one, it is essential to foster a culture where employees share knowledge and expertise, and are also encouraged to learn, especially from past experiences. An organizational culture that fosters knowledge management supports the organization’s ability to keep pace and adapt to the changing needs of its environment while maintaining its relevance and competitiveness [9]. Preserving continuity of knowledge and operations is a significant challenge for organizations; they need to master their business history lest the quality of service and products be impaired. However, losing unique knowledge due to a specific employee’s departure can reduce the quality and efficiency of the whole organization. “Departure” refers to (1) scheduled departure due to an age limit such as the legal retirement age; (2) transfer to another position as part of a promotion or re-organization; (3) employee or management decision to terminate the contractual relationship before the legal retirement age, regardless of the reason. Knowledge continuity management helps overcome this drawback by enhancing quality management of organizational and business processes, chiefly for knowledge-based processes, leading to management improvement [10].

Successful “knowledge management” and “knowledge continuity management” require a paradigm change in corporate culture and adaptation that encourages creating, sharing, and distributing knowledge within the organization [11]. The US aerospace manufacturing industry has experimented with this “change of mind” by encouraging managers to understand and reduce the knowledge gap. Their challenge was to develop competitive advantages by developing a knowledge-sharing culture driven by multigenerational legacy knowledge, transforming manufacturing workers into highly skilled professionals [12]. Successful and efficient knowledge management (KM) initiatives rely on three main kinds of critical organizational infrastructures: knowledge culture (KC), organizational structure, and knowledge technology [13].

This search for the “holy grail” leads management to focus on technological and managerial changes that can improve the load on the workforce confronted with the complex perspectives of globalization and growing market competition. Therefore, knowledge management and knowledge continuity management are ubiquitous learning approaches and the new pillars that support the business value of any organization.

In recent decades, the development of information systems–computerized and computational technologies address data, information, and knowledge in all forms–has contributed to enhancing capabilities across the entire knowledge management landscape, becoming a significant element in a paradigm change. However, a vital component of an organization’s knowledge is tacit and consists of unwritten or verbal instructions and procedures [14, 15]. Encouraging the conversion of this tacit content into explicit knowledge shared in a formal, systematic, and measurable way is a crucial challenge [16–18]. Organizational knowledge, both tacit and explicit, is fundamental to organizational culture and critical for its ability and capacity to innovate and adapt to market changes. Staff members on the individual level decide whether they will use tacit knowledge. Therefore, they use computerization and computational technologies that enable efficient and effective knowledge management, and knowledge continuity management systems, which are scalable to workplace environments [19–21].

From the perspective of a business and its competitiveness, knowledge management is a primary asset that can be leveraged to attain excellence and attract investors. Consequently, using knowledge management systems based on accepted standards, such as the one developed and maintained by the International Organization for Standardization (ISO) ISO 30401, are strategic blocks for building a solid, persistent reputation by “establishing, implementing, maintaining, reviewing and improving an effective management system for knowledge management in organizations” [22, 23]. Furthermore, the information security dimension of knowledge management is also a matter of great interest and a critical focal point. The relevant standard is ISO 27001 [24], which focuses on information security and plays a strategic role in guiding organizations in “establishing, implementing, maintaining and continually improving an information security management system.” It includes requirements for assessing and treating information security risks and is well-known throughout the defense industry. As with any general standard, its guidance is generic and implementable in any organization regardless of type, size, or nature [25, 26]. ISO 27001 encourages users to deal with knowledge management safety and retention to ensure information systems security. This goal can be met using technological components and human-oriented actions to enhance employees’ information security awareness [27].

Total Quality Management (TQM) was an integrative framework considered a pillar supporting organizations for several decades. Combined with ongoing knowledge management, TQM enhances an organization’s ability to produce and share new knowledge with its entire workforce. This approach helps increase competitive advantage and overall performance to attain world-class status. Nevertheless, these improvements are possible only if the whole organization is actively engaged in knowledge management objectives as part of quality management. Moreover, the entire staff must be responsible for achieving efficiency improvements at the individual level, as suggested by the seven quality management principles and the ISO 9000 family of standards, which are (a) customer focus, (b) leadership, (c) engagement of people, (d) process approach, (e) improvement, (f) evidence-based decision making, and (g) relationship management [28]. Therefore, previous empirical studies have confirmed that effective implementation of TQM can lead to improved performance [29–32].

Knowledge retention and transfer are pillars of knowledge management within an organization’s quality management process. Indeed, having an organizational memory is essential for understanding and learning from mistakes, failures, and any kind of crisis [33, 34].

Knowledge retention and organizational memory are similar but deal with different scales. Organizational memory focuses on all data, information, and knowledge acquisition, storing, and retrieval in the entire organization daily. Conversely, the knowledge retention process is dynamic. It aims to preserve crucial intellectual resources for business continuity as a countermeasure of the permanent knowledge memory loss (e.g., employee’s retirement or resignation). Knowledge sharing is the applicative aim of the knowledge memory and an organizational asset that enables knowledge development and innovation to support the organization’s market competitiveness. Practically, knowledge sharing is a learning process. Each staff member in an organization gains knowledge from others and shares it with them. Different methods, systems and technological platforms support knowledge sharing as a part of knowledge retention, depending on the business field (e.g., defense industry or high-tech) [35–37].

Many companies have a workforce representing three and even four generations [38]. Severe, global financial and economic crises (e.g., the COVID-19 pandemic) have many and varied repercussions, including both unemployment and the need to delay departure; they may force employees to continue working for more years than they initially planned but also enhance the overall job engagement and knowledge-sharing behavior [39]. This means that multiple “workforce generations” are employed simultaneously in a workplace, obliging employers to build and operate “training systems” suited to the needs and characteristics of each generation [40]. The varied characteristics, work ethics, core values, and methods of communication and learning characterizing each generation [41, 42] influence workflow processes. Developing personalized knowledge retention and transfer tools is fundamental for supporting efficient and effective productivity in a healthy work environment [43].

### Aim, objective, and hypotheses

The present study aims to provide decision-makers in the defense industry with tools to develop and implement knowledge retention and transfer policies before an employee departs. Based on prior theoretical and applicative research highlighting that organization knowledge sharing is led by factors related to interpersonal (i.e., between employees and management) trust and communication, e.g., social identification with the organization [44, 45], our principal objective is to understand knowledge retention and transfer processes between workforce generations in a cutting-edge Israeli industry. We documented the dramatic losses of knowledge caused by employees’ departure. We propose suitable mechanisms that could be implemented to create a suitable knowledge retention system. Three hypotheses led our research:

1. Multigenerational knowledge sharing can improve a cutting-edge industry (e.g., defense), by reducing knowledge loss.
2. Enhancing multigenerational learning and knowledge retention systems requires changes in the quality systems of a cutting-edge industry.
3. Improving the multigenerational knowledge-sharing process must be based on methods and systems suggested by ISO standards for facilitating short, mid, and long-term resilience.

After introducing the challenges and our hypotheses, we describe our data sources and our study’s qualitative and quantitative methodologies below before discussing the analysis results.

## Material and methods

This two-stage study examined knowledge retention processes in a cutting-edge Israeli industry (e.g., defense). The first stage was qualitative, exploratory research based upon semi-structured interviews, followed by quantitative research using a questionnaire (S1 File) in the second stage. The questionnaire asked about the changes that would be necessary for the companies’ quality systems to enhance multigenerational learning and knowledge preservation [46, 47].

The overall process included the following steps:

- Qualitative research:
  ◦ Exploratory study to investigate a problem that has not been studied or thoroughly investigated in the past.
  ◦ Triangulation to increase the credibility and validity of research findings.
- Quantitative research:
  ◦ Questionnaire creation, validation, and distribution to relevant professionals.
  ◦ Data analysis: The data received in response to the questionnaire were analyzed for two main subjects:
    1. Relationships between the characteristics of the participants (seniority and education) and knowledge management.
    2. Professional reference to knowledge management in the organization.
  Suggestions for updating the knowledge retention policy.

The questionnaire was reviewed and approved by the ethics committee of the Faculty of Industrial Engineering and Management of the Holon Institute of Technology (TM/2/2020/SK/001). The information provided by the participants during the distribution of the questionnaire is stored in a secured, encrypted manner, with access restriction services provided by one of the researcher’s (SK) institution.

### Qualitative research

The qualitative research included two sub-stages, semi-structured interviews, and triangulation.

The semi-structured interviews were designed to help researchers understand the general approach to knowledge management within the organization [48]. The interviews were conducted privately and consisted of six questions designed to collect data as a foundation for constructing the qualitative questionnaire. The interviews have also designed to indicate the relevance and appropriateness of the research questions and avoid collecting useless data. This process contributes to the study’s credibility by providing additional points of view. Due to the work environment in the defense field, all five people selected to be interviewed hold senior positions and have ample experience, with more than 20 years of seniority. All interviewees consented to be interviewed for research purposes [49]. All were asked the same six foundational questions (Table 1). Some interviews also included supporting questions as part of the dynamic developed during the process. The interviews were fully recorded and later transcribed verbatim.

**Table 1:** Summary of the answers given by interviewees.

Question	Answers
From your knowledge of the organization, do you feel there are resources allocated to transferring knowledge from employees with years of professional experience to younger employees?	From the answers to this question, it appears that allocating resources for knowledge transfer does not receive the required managerial attention. Furthermore, all five interviewees mentioned that the need for management to allocate resources and transfer knowledge arose only in the past year.
Do you think support software for knowledge management, such as artificial intelligence, should be used?	In their responses, all five interviewees agreed that there is a need for support software that would make it easier to find information and compile it into a central database.
Would an organized procedure concerning subjects to transfer and a built-in mechanism (technological tools) serve the purpose, or would an in-person meeting be preferred?	All five interviewees agreed that an organized procedure for departing employees should be prepared and implemented to ensure the conservation of rare and vital knowledge.
Experienced employees acquire tacit knowledge, the internal knowledge, that they acquire through their work experience. How can this knowledge be transferred, in your opinion?	The answers regarding the transfer of knowledge from experienced employees to the next generation can be divided into a few categories: using documentation and a supportive organizational culture; establishing knowledge groups for mutual enrichment and the exchange of ideas; and an organized mentoring program.
How do you conserve your knowledge? What tools do you use?	The interviewees’ answers varied. Each one embraced a different method of knowledge conservation: organized training with presentations, informal transfer, and retaining the knowledge in computer files. It seems that this important task requires an organized procedure.
Are there any other issues that you wish to add which were not mentioned here?	All five interviewees agreed that knowledge retention is necessary and that organizational culture is critical for successful knowledge transfer.

In qualitative research, triangulation is a method for cross-referencing data from trustworthy sources, and basing findings on at least three different independent information sources. It examines a phenomenon from different perspectives and therefore provides more accurate observations of the subject. We summarized the main points raised in each interview and then compared them. The triangulated findings were the leading criteria for constructing the questionnaire [50].

### Quantitative research

The quantitative stage was based upon a questionnaire for data collection, allowing us to examine (a) intergenerational knowledge management and retention within the participants’ organizations and (b) the impact of the quality systems on knowledge preservation. The questionnaire reflects the leading criteria that arose from prior analyses reported in the literature, the semi-structured interviews, and the triangulation: procedures, the administration’s commitment, knowledge management indices, willingness to change, and reference to transferring tacit knowledge. The questionnaire included two sections:

1. Four socio-demographic questions (i.e., age, education, professional experience, industrial field, and knowledge conservation and management) [51].
2. Questions related to knowledge management, sharing, and retention:
  - A set of 18 questions to be answered on a five-point Likert scale [52] (S1 File) focusing on the level of their agreement (i.e., Absolutely disagree, Disagree, Undecided, Agree, Absolutely agree) with the statements about knowledge management and retention activity in the organization. The statements concerned (a) Knowledge management and knowledge transfer structure in the organization; (b) Knowledge management control in the organization; (c) Knowledge creation in the organization; (d) Knowledge sharing in the organization; (e) Level of mentoring in the organization; (f) Level of participation in conferences in the organization.
  - A set of 5 multiple-choice, closed questions related to the management’s commitment to the issue of knowledge retention and management.

Three experts in the field validated the questionnaire ‒ two academics and one from the industry–to ensure that the questions were phrased clearly, and that the questionnaire evaluates relevant details [53]. The experts were asked to rate each question for clarity and relevance to the subject, using a Likert scale: 1– very low to 5 –very high. Space was also provided for additional notes. The questionnaire was then revised following the experts’ comments.

Google Forms was used to distribute, by email, the final questionnaire to a sample of 90 potential respondents who were selected using convenience sampling. Of this group, 81 completed the questionnaire between October 10 and November 9, 2020. The respondents were all employed in engineering and development and had a bachelor’s degree or higher. These employees possess knowledge that is the most vital and critical for the organization. The sample consisted of 56 men and 25 women, accurately reflecting the gender ratio in the industry. Once the questionnaires were completed, the responses were analyzed.

## Results

### Qualitative research and triangulation

In the qualitative, exploratory phase, we collected various points of view on the subject using semi-structured interviews. The questions asked and the answers provided by the interviewees are summarized in Table 1.

### Quantitative questionnaire

Responses to the questionnaire were analyzed using several statistical tests. We examined the extent of the correlations between the evaluation of criteria for improving multigenerational learning and reducing knowledge loss and between the changes in the quality systems and enhancing multigenerational learning.

#### Multigenerational learning and knowledge loss

The first analysis examined how multigenerational learning can be improved and knowledge loss reduced. Linear regression evaluated the correlations between statements related to these issues. Significant correlations were found in two instances.

A significant correlation (P<0.05, R^2^ = 0.48) was found between statements about the organization’s level of encouragement (S1 File, question 15: “Does the organization you work for encourage the learning of new methods and systems to improve organizational knowledge?”) and the frequency of knowledge management (S1 File, question 21: “How would you rate the ability of your department/project to manage the knowledge they have accumulated over the years?”). Similarly, the correlation between the organization’s level of encouragement and the frequency of participation in conferences (S1 File, question 20: How often do you attend professional conferences?) is also relatively high and significant (P<0.05, R^2^ = 0.61).

#### Changes in quality systems

The second analysis examined the changes required in the quality systems of companies in cutting-edge industries to enhance multigenerational learning and the conservation of multigenerational knowledge. One-way analysis of variance (ANOVA) [54] was used to evaluate significant differences between participants with different amounts of professional experience and their frequency of involvement in knowledge management and departmental attention.

The results show significant differences between professional experience and frequency of attention to knowledge management (P<0.05). Table 2 shows that the average frequency of knowledge management among most participants with many years of professional experience (Mean = 2.2, SD = 1.1) is significantly higher than participants with fewer years of professional experience (Mean = 2.2, SD = 1.03). The results for employees with five or fewer years of seniority might not accurately reflect the correlation due to the small number of participants in the sample.

**Table 2:** One-way ANOVA for differences in professional experience in the organization and the frequency of knowledge management and departmental attention.

Seniority (in years)	N	Mean	SD
Less than 1	2	2.50	0.71
1–5	6	2.67	0.52
6–10	10	2.20	1.03
10–20	35	2.94	1.00
20–35	28	3.43	1.17

ANOVA was also used to examine whether there is a difference between participants with different amounts of professional experience and their improvement in knowledge management. The analysis showed significant differences between the number of years of professional experience and the frequency of knowledge management (P<0.05). Table 3 shows the average response to the question, “Can you improve or promote the issue of knowledge management on a personal level?” for participants with many years (more than 20 years) of professional experience (Mean = 3.89, SD = 0.83) is significantly higher than for participants with fewer years (less than five years) of professional experience (Mean = 2.66, SD = 0.51). The results for employees with one year of seniority might not accurately reflect the correlation due to the small number of participants in the sample.

**Table 3:** One-way ANOVA for differences in professional experience in the organization and improvement of knowledge management on a personal level.

Seniority (in years)	N	Mean	SD
Less than 1	2	3.50	0.71
1–5	6	2.67	0.52
6–10	10	3.30	0.95
10–20	35	3.54	0.95
20–35	28	3.89	0.83

In a multivariate regression analysis, we evaluated the relative contribution of three parameters: (1) encouraging learning in the organization and improvement of knowledge management systems; (2) the frequency of organizational attention to knowledge management; and (3) familiarity with the concept of knowledge management. Table 4 presents the results of the regression. It shows that the variance explained by the variable: “Frequency of organizational attention to knowledge management” is significant (P<0.001, R^2^ = 0.48). However, only the variable: “Familiarity with the term knowledge management and its practical application at work” significantly contributes to the explained variance. Furthermore, when familiarity with the concept and its implementation increases, so does the frequency of attention to knowledge management and the encouragement to learn and improve this field.

**Table 4:** One-way ANOVA for differences in professional experience in the organization and improvement of knowledge management on a personal level.

Question	Coef	SE Coef	T-Value	P-Value
Constant	0.66	0.48	1.37	0.17
Does the organization encourage learning?	0.49	0.10	5.07	0.00
Familiarity with the concept of KM				
Yes, I have heard the term	0.73	0.42	1.76	0.08
Yes, I know the term and apply it in my work	1.60	0.43	3.72	<0.001

An additional multivariate regression analysis was conducted to find the possible correlation between the ranking of the department’s or project’s ability to manage their accumulated knowledge, ensuring that professional knowledge is documented, and the existence of a dedicated documentation system. We examined the correlation between “Documentation of professional knowledge,” “Ranking of the department that documents professional knowledge,” and “Existence of a documentation system in the organization.” This one is significant (P<0.05, R^2^ = 0.28). Moreover, when there is no documentation system in the company, the department’s or project’s ability to manage the accumulated knowledge (Coef = -0.34) decreases. Furthermore, we also pointed out that when there is a documentation system in the company, the workers’ ability to manage the accumulated knowledge (Coef = 0.31) increases. Meaning if a documentation system is lacking, the department or project’s documentation ability will be low (Table 5).

**Table 5:** Output results of multivariate regression analysis.

Question	Coef	SE Coef	T-Value	P-Value
Are you careful to document (Yes/No)?	1.279	0.18	7.03	<0.01
Is there a system in the organization for documenting new processes?	0.12	0.05	2.64	0.01
No	-0.34	0.17	-1.97	0.00
Yes	0.31	0.11	2.83	0.01

The main conclusion that can be drawn from this analysis is, unsurprisingly, that documentation management capabilities are lower in companies that lack a proper documentation system. Accordingly, companies, particularly in the defense industry, need to develop or implement well-designated documentation systems to increase their ability to manage the conservation of knowledge. It is inadequate to entrust this task to positive and caring people, leaving them to implement it independently without clear procedures and a support system.

## Discussion

### Principal findings

Our main objective was to understand the factors influencing the development of an organizational culture encouraging innovation, knowledge sharing, organizational learning, openness, and providing opportunities for creating new knowledge. Therefore, we used several quantitative and qualitative tools to assess our research hypotheses from different perspectives.

The qualitative research yielded an extensive list for preserving and managing organizational knowledge. The criteria mentioned most frequently in the interviews were: “familiarity with the subject of knowledge management,” “knowledge sharing policy,” “encourage learning new systems,” and “addressing tacit knowledge.” A few additional criteria arose from the literature on knowledge retention and management, tacit and explicit knowledge types [55], and references to “generation gaps” (generation X and generation Y).

Respondents were asked to rank these criteria to confirm or refute our hypothesis about the connection between improving multigenerational learning and reducing knowledge loss. The two types of qualitative analysis were run. In the first analysis, the variable was entered as an interval scale variable. The second variable was entered as a categorical variable on an ordinal scale. The results showed that when we used a categorical variable on an ordinal scale, the variance explained by the dependent variable was higher.

The descriptive statistics regarding knowledge management characteristics highlighted that:

- 50.6% (41/81) of the interviewees had heard of the term “knowledge management” but reported that it is not implemented in the organization.
- 53.1% (43/81) responded that the management does not emphasize knowledge management.
- 88.8% (72/81) agree that knowledge management is essential and should be implemented within the organization.
- 85.2% (69/81) agree that adopting a knowledge management policy is essential for the organization.
- 73.8% (59/81) stated that knowledge management requires improvement.

The findings, as mentioned earlier, are consistent with the previously reported research on knowledge management as a structured, consistent, and methodical process that facilitates the complicated task of creating, managing, and transferring knowledge within an organization. We note that 50.6% (41/81) feel social support for sharing knowledge, and 95.1% (77/81) of the interviewees claimed that tacit knowledge is transmitted and not intentionally withheld. Tacit knowledge is currently perceived as critical knowledge in the organization because experts can achieve significant time-saving improvements [56]. Most of the survey participants replied that the departure of employees who are considered vital sources of knowledge requires preparation on the organizational level using an organized, structured plan. This is consistent with the literature regarding the culture of knowledge sharing and multigenerational influences. The oldest or “silent” generation is considered a source of knowledge; it is characterized by loyalty to the system and obedient employees [57]. This is also true of the “Baby Boom” generation, who are very loyal and devoted to their work. These generations are considered sources of knowledge in the organization. In contrast, the younger Y generation is characterized by a high level of technical knowledge as well as the ability to multitask. This generation prefers working as a team over independent work and prioritizes cooperation and teamwork. Understanding the characteristics of each generation, and the need to address them in their own language, helps resolve intergenerational conflicts [58].

The “level of encouragement to learn knowledge management systems and familiarity with them” and “frequency of knowledge management in the organization” are essential for improving multigenerational learning and reducing knowledge loss (P<0.001, R^2^ = 0.30). Moreover, “ranking of the department’s ability to manage their accumulated knowledge” and “making sure that professional knowledge is documented” are significantly correlated (P<0.01, R^2^ = 0.078). This link points out the importance of efficiently managing historical knowledge.

Another critical criterion for conserving multigenerational knowledge is familiarization with organizational knowledge sources, which can contribute to the transfer of multigenerational knowledge. Multivariate regression analysis was performed on the criteria that are significant for organizational knowledge conservation: “Does the organization encourage learning new methods and improving the knowledge systems?” and “organizational frequency and attention to knowledge management” (R^2^ = 0.48).

The quantitative analysis clearly shows that some criteria are more important than others, and some carry greater weight in the final calculation than others. The findings of the quantitative analysis confirmed the first research hypothesis. It should be noted that the findings from the quantitative analysis and the findings from the qualitative analysis were congruent. There is a positive correlation between improving multigenerational learning and reducing knowledge loss.

Our second hypothesis focused on the relations between changes in the quality systems and enhancing multigenerational learning. The findings from the qualitative analysis yielded several criteria for addressing the improvement of knowledge management in terms of quality. The criteria which appeared in most of the interviews were: “Knowledge quality management,” “TQM,” “Procedure control,” and “Managerial indices”.

The descriptive statistics yielded the following results:

- 82.7% (67/81) of the respondents support managerial policies and indices regarding knowledge management.
- 60.5% (49/81) are familiar with the process documentation system in the organization. However, only 55.5% (45/81) ensure that they document knowledge in the organization, meaning 44.4% (36/81) of the respondents are not careful about documenting knowledge.
- 50.6% (41/81) of the respondents mentioned that periodic inspections of knowledge management are not conducted in the organization.

Links between knowledge management and TQM have been previously defined in industries (excluding “defense”) [59]. Specifically, when associated with knowledge management processes, TQM practices are an essential source of competitive advantage because they can promote knowledge sharing between the organization’s staff members. Therefore, the support and commitment of senior management are required. Leadership commitment consists of developing and implementing an environment that responsibly empowers people to create valuable knowledge.

The criteria “Every organization should have managerial indices” and “Do you know any highly experienced sources of knowledge in this organization?” are significantly correlated (P<0.001, R^2^ = 0.10), meaning that understanding the need for management metrics for knowledge management is connected to familiarity with the knowledge centers in the organization.

The variance analysis showed significant differences between professional experience and frequency of attention to knowledge management (P = 0.003). Additionally, we noticed significant differences between years of professional experience and personal improvement regarding knowledge management (P = 0.004).

The statistical findings indicate that managerial leadership is required for knowledge management and an ongoing control and support system. The findings were consistent with prior studies unrelated to knowledge retention in the defense industry regarding the increasing importance of knowledge management.

### Limitations

The study sample, including 81 managers and engineers, was drawn from cutting-edge industries (mainly in the defense field) in Israel. We recommend broadening the study by using a larger sample to improve the statistical validity for more reliable and comprehensive results. Further research should also interview experts in additional academic and industrial fields. Moreover, it would be interesting to identify and analyze differences in the knowledge retention culture between subfields within the defense industry (e.g., aerial systems, ground systems). The sample could be expanded into other industries and examine diverse populations from different organizational cultures and fields.

### Future perspectives

This study was motivated by a wish to help decision-makers develop an organizational culture that encourages innovation, cultivating a knowledge-sharing environment, organizational learning, an open atmosphere, and opportunities for creating new knowledge. The current findings present several critical parameters for the success of multigenerational knowledge retention and the changes required in the organizational quality system. First, it is necessary to determine a knowledge management strategy according to ISO 30401, which defines how an organization should manage its knowledge. Then, administrative and professional responsibility for promoting knowledge management activity should be assigned. The organization needs to inculcate knowledge management values in employees and provide ongoing support systems. Finally, quality monitoring scores should be established to ensure the implementation of the knowledge management system.

Knowledge management must be implemented as an integral part of the organizational procedures, improving them to enhance the organizational achievements. It revolves around people and the knowledge and experience accumulated over time to promote a culture of sharing and learning within the organization. Supportive technological systems assist in implementing knowledge management procedures, such as storing the knowledge in an easily retrievable form and using virtual sharing systems. Therefore, standards such as ISO 30401 set requirements for a knowledge management system in organizations, covering the establishment and maintenance of knowledge management systems, the implementation of knowledge management and sharing culture, knowledge management solutions, and ways to measure knowledge in the organization. Accordingly, from a managerial perspective, the main implication of this study is that we highlighted the need for cutting-edge technology businesses to develop internal policies based on the current standard. This must stimulate the development and continuous improvement of employee-targeted communications supporting individual and collective knowledge management capacities. From a theoretical perspective, this implies that large-scale studies must be run in cutting-edge industries to understand and support the development of domain-specific knowledge management policies.

## Conclusion

Retaining knowledge retention and preserving operational continuity is a significant challenge for organizations, specifically in the highly competitive defense industry. The potential damage to the quality of service and products delivered to the client due to loss of exclusive knowledge caused by employees’ departure can significantly impact competitiveness and financial results.

The focus of this study was to assess the changes required in the quality systems of companies in the defense industry to enhance multigenerational learning and knowledge conservation. In addition, we examined the extent of the correlation between the evaluation criteria for improving multigenerational learning and reducing knowledge loss and the extent of the correlation between the changes required in the quality systems of defense companies to enhance multigenerational learning and conserve multigenerational knowledge.

The approach to knowledge management is outlined, by default, in the quality management standards. These require conservation of the knowledge accumulated in the organization’s core processes and quality records–analysis of complaints, client surveys, tests, improvement processes–but not for a written procedure. The “knowledge management systems” standard encourages and focuses on a procedure for managing organizational knowledge, but the standard for managing “information systems security” is also essential. It sets requirements for establishing, implementing, maintaining, and continuously improving an information security management system to help organizations make their knowledge assets safer.

Our qualitative study asked whether the company could transfer knowledge from the older generation to the younger generation, which applies to all employees. Analysis of the qualitative and quantitative findings showed that the importance of knowledge management is acknowledged and that there is a need for a supportive organizational culture to transfer knowledge successfully. Analysis of the quantitative findings clearly shows that the respondents believe that some specific criteria are more critical than others.

## Supporting information

S1 File Questionnaire. Questionnaire about the changes that would be necessary for the companies’ quality systems to enhance multigenerational learning and knowledge preservation.
(PDF)
