# Supplementary material for: Intergenerational knowledge management in a cutting-edge Israeli industry: Visions and challenges
Source: PLoS One. 2022 Jul 8;17(7):e0269945. doi: 10.1371/journal.pone.0269945 (PMC9269463; doi:10.1371/journal.pone.0269945)
Supplement: S1 File — Questionnaire about the changes that would be necessary for the companies’ quality systems to enhance multigenerational learning and knowledge preservation. (PDF) [file pone.0269945.s001.pdf]

S1 File: Questionnaire

Socio-demographics-related questions

**No.**	**Question**
1	Gender
2	Age
3	What is your education?
4	Several years of experience in the organization

Likert-scale questions

**No.**	**Question**
5	I know the term “knowledge management” and apply it regularly at work.
6	Knowledge management in projects is managed understandably.
7	Every employee is an investment of the organization. Do you agree that the knowledge he has gained also belongs to the organization? Must therefore share it
8	Every organization should have managerial metrics. Do you think KM should be a part of them?
9	Do you take care to document the professional knowledge you have gained as part of your work?
10	Do you know employees of knowledge centers who have expertise in your organization?
11	In the department/organization where you work, is there a periodic audit to present KM? View report folders/version management for drawings and the like
12	Do you agree with the statement: It is important to refine and assimilate the issue of KM in the department/organization?
13	Do you agree with the statement: Organizations must be constantly updated and acquire new and relevant knowledge?
14	Can you improve/promote on a personal level the issues related to knowledge management and retention to help the organization improve itself?
15	Does the organization you work for encourage learning new methods and systems to improve organizational knowledge?
16	Is there a system in the organization for documenting new processes/drawings that can be used for significantly similar projects in the future?
17	Do you feel that the organization in which you work has social support (knowledge transfer between colleagues) that contributes to information and knowledge sharing?
18	What do you think about the possible adoption of a knowledge-sharing policy in the organization?
19	Is it true that much information is deliberately kept quiet with colleagues and thus makes them knowledge centers and necessary for the organization?
20	How often do you attend professional conferences?
21	How would you rate the ability of your department/project to manage the knowledge they have accumulated over the years?
22	My mentor/supervisor intervenes in explaining the work processes/tutoring understandably and fluently.

Multiple-choice closed-ended questions related to KM

No.	Question
23	What activities will be performed in your department/project that you think will help in KM?
24	After retiring/leaving knowledge centers in the organization, do you think the organization can function without any problems?
25	What do you think are the challenges in acquiring knowledge?
26	It is not easy to share the knowledge I have with colleagues because:
27	How often would you recommend managing the professional knowledge you have gained?
